# Cardiovascular disease in Alpha 1 antitrypsin deficiency: an observational study assessing the role of neutrophil proteinase activity and the suitability of validated screening tools

**DOI:** 10.1186/s13023-024-03124-x

**Published:** 2024-03-21

**Authors:** E. Sapey, L. E. Crowley, R. G. Edgar, D. Griffiths, S. Samanta, H. Crisford, C. E. Bolton, J. R. Hurst, R. A. Stockley

**Affiliations:** 1 Institute of Inflammation and Ageing, University of Birmingham, Birmingham, B15 2GW UK; 2 University Hospitals Birmingham NHS Foundation Trust, Edgbaston, Birmingham, West Midlands UK; 3 Institute of Applied Health, University of Birmingham, Birmingham, West Midlands UK; 4 UCL Respiratory, University College London, London, UK; 5 NIHR Nottingham BRC Respiratory Theme, School of Medicine, University of Nottingham, City Hospital NUH Trust, Nottingham, UK

**Keywords:** Cardiovascular disease, Chronic obstructive pulmonary disease, Emphysema, Alpha 1 anti-trypsin deficiency, Arterial stiffness, Risk assessment, Neutrophils

## Abstract

**Background:**

Alpha 1 Antitrypsin Deficiency (AATD) is a rare, inherited lung disease which shares features with Chronic Obstructive Pulmonary Disease (COPD) but has a greater burden of proteinase related tissue damage. These proteinases are associated with cardiovascular disease (CVD) in the general population. It is unclear whether patients with AATD have a greater risk of CVD compared to usual COPD, how best to screen for this, and whether neutrophil proteinases are implicated in AATD-associated CVD. This study had three aims. To compare CVD risk in never-augmented AATD patients to non-AATD COPD and healthy controls (HC). To assess relationships between CVD risk and lung physiology. To determine if neutrophil proteinase activity was associated with CVD risk in AATD. Cardiovascular risk was assessed by QRISK2® score and aortic stiffness measurements using carotid-femoral (aortic) pulse wave velocity (aPWV). Medical history, computed tomography scans and post-bronchodilator lung function parameters were reviewed. Systemic proteinase 3 activity was measured. Patients were followed for 4 years, to assess CVD development.

**Results:**

228 patients with AATD, 50 with non-AATD COPD and 51 healthy controls were recruited. In all COPD and HC participants, QRISK2® and aPWV gave concordant results (with both measures either high or in the normal range). This was not the case in AATD. Once aPWV was adjusted for age and smoking history, aPWV was highest and QRISK2® lowest in AATD patients compared to the COPD or HC participants. Higher aPWV was associated with impairments in lung physiology, the presence of emphysema on CT scan and proteinase 3 activity following adjustment for age, smoking status and traditional CVD risk factors (using QRISK2® scores) in AATD. There were no such relationships with QRISK2® in AATD. AATD patients with confirmed CVD at four-year follow up had a higher aPWV but not QRISK2® at baseline assessment.

**Conclusion:**

aPWV measured CVD risk is elevated in AATD. This risk is not captured by QRISK2®. There is a relationship between aPWV, lung disease and proteinase-3 activity. Proteinase-driven breakdown of elastin fibres in large arteries and lungs is a putative mechanism and forms a potential therapeutic target for CVD in AATD.

## Background

Alpha 1 Anti-trypsin deficiency (AATD) is a rare, genetic cause of Chronic Obstructive Pulmonary Disease (COPD) with an emphysema dominant phenotype. It affects between 1 in 2000 to 1 in 5000 individuals [1]. The lung disease associated with AATD is variable but in general, is associated with faster progression and decline in lung physiology compared with non-AATD COPD [2]. The causal association between neutrophil proteinase activity and AATD-associated lung disease is well described [3], and lung disease can be slowed using Alpha 1 Antitrypsin (AAT) augmentation therapy, replacing the deficient anti-proteinase via intravenous infusion [2], [4]. AAT augmentation therapy is not available in all countries, and many patients are managed according to usual COPD guidelines.

It is increasingly recognised that patients with AATD also experience other diseases commonly associated with COPD, such as periodontitis [5]. Recognising co-morbidities enables a more holistic approach to patient management and also offers opportunities to identify common biological mechanisms across diseases which might be therapeutically targetable.

Cardiovascular Disease (CVD) is a common co-morbidity in non-AATD COPD [6], sharing the risk factors of smoking cigarettes, poverty and a sedentary lifestyle. In non-AATD COPD, CVD can be predicted non-invasively using tools such as the QRISK2® score [7], which is a composite score including age, sex, ethnicity, zip/postcode (as a surrogate marker of social deprivation), smoking status, BMI, the presence of other key CVD-associated co-morbidities (diabetes, hypertension, chronic kidney disease and atrial fibrillation) and CVD in a first degree relative [8]. CVD risk can also be assessed directly using physiological measurements including arterial stiffness. Aortic stiffness reflects the pathological state of central arteries and is associated with coronary artery atheroma burden [9]. It is measured using carotid-femoral (aortic) pulse wave velocity (aPWV) [10]. There is concordance between QRISK2® and arterial stiffness in non-AATD COPD [11], with high QRISK2® being associated with high arterial stiffness. There are also weak relationships between cardiac risk using either score or measure and the severity of lung disease in COPD [12], [13].

It is unclear whether patients with AATD are also at greater risk of CVD than the general population. The potential confounders such as socio-behavioural factors associated with CVD (smoking and poor socioeconomic status) are less prevalent in patients with severe AATD [14] and COPD presents at a younger age enabling the relationship to COPD to be studied with less effect of confounders. The studies available of CVD risk in AATD have provided conflicting results with no mechanism of effect suggested [15]–[20]. Three small studies have noted that AATD patients have increased aPWV compared to controls [16], [21], [22]. However, a metanalysis of five studies suggested that AATD patients have a lower risk of ischaemic heart disease [20]. The UK Biobank similarly observed that patients with Z allele heterozygosity have a lower risk of cardiovascular disease than wild type although the risk in those with Z allele homozygosity was not described [18]. However, there is a strong rationale for assuming an increased susceptibility to CVD in AATD. The neutrophil proteinases so intrinsically linked with lung disease in AATD are also implicated in CVD, associated with impaired microvascular perfusion, left ventricular dilatation and adverse cardiac events [23]. Proteinase 3 (a neutrophil proteinase) is increased acutely and predicts outcomes following myocardial damage [23], [24] and is associated with general cardiovascular risk [25].

Because of AAT deficiency neutrophil proteinases may also contribute to both lung and CVD disease. Were this the case, any relationships would be most apparent in AATD patients who were not receiving AAT augmentation therapy, as neutrophil proteinase activity is much higher in AATD compared to non-AATD COPD [26] and AAT augmentation therapy reduces neutrophil proteinase activity [27]. Additionally, were neutrophil proteinases more implicated in the pathogenesis of CVD in AATD than classical risk factors (such as smoking, poverty and certain conditions such as diabetes), standard screening tools such as QRISK2® maybe less able to detect CVD risk in this rare disease.

We hypothesised that CVD risk would be elevated in AATD compared to healthy controls and non-AATD COPD, but less detected using the standard screening tool, QRISK2®. Further, the elevated CVD risk in AATD would be associated with unopposed neutrophilic inflammation in AATD, with relationships between CVD, lung disease and neutrophil proteinase activity.

The purpose of the present study was threefold.

First, to investigate the risk of CVD in a cohort of never-augmented AATD patients, non-AATD COPD and healthy controls using both a calculated risk score (QRISK2®**)** and aPWV, including which aspects of the composite scoring for QRISK2® contributed to risk in each group.

Second, to determine the relationships between CVD risk measures and lung physiology in patients with AATD.

Third, to determine if proteinase 3 activity was associated with CVD risk in patients with AATD.

## Results

### Cohort demographics

We recruited 228 patients with AATD, of whom 221 were PiZ and 7 were Z null genotypes; 180 of these were recruited from the University Hospitals Birmingham NHS Foundation Trust and 48 were recruited from the Royal Free London NHS Foundation Trust. These were matched physiologically to 50 patients with non-AATD COPD. Sex was matched across all groups. 51 healthy controls (HCs) were matched for age with non-AATD COPD group. Participant demographics are described in Table 1. There were no differences between groups in the prevalence of the following co-morbidities; diabetes, chronic kidney disease, atrial fibrillation, rheumatoid arthritis, treated hypertension. See Table 2.

**Table 1:** Participant demographics

	AATD	Non-AATD COPD	Healthy controls	P value
Number	228	50	51	
Sex (n male: female)	144:84	33: 17	28:23	0.4013
Age (years), median (IQR)	**58 (48**–**65)**	**71 (67**–**77)**	**68 (60–77)**	< 0.0001*
PiZ	221	0	0	
PiZ null	7	N/A	N/A	
Blood AAT concentration Median (IQR)	4.1μM (3.0–6.2)	26.1 mM (21.3–29.0)	22.3 mM (19.8–26.3)	
Never smoked, n (%)	**58 (25.4%)**	**0**	**9 (17.6%)**	0.0003**
Ex-smoker, n (%)	165 (72.4%)	40 (80%)	38 (74.5%)	0.213**
Current smoker, n	**5 (2.2%)**	**10 (20%)**	**4 (7.8%)**	0.008**
Pack year history. Median (IQR)	**12 (5–21)**	**44 (34–59)**	**21 (12–35)**	< 0.0001*
FEV_1_ (L) median (IQR)	**1.6 (1.1–2.3)**	**1.3 (0.9–1.8)**	**2.8 (2.3–3.4)**	< 0.0001*
FEV_1_ (percent predicted). Median (IQR)	**51.7 (36.2–81.3)**	**49.5 (40.0–72.0)**	**101 (92.0–112.5)**	< 0.0001*
FEV_1_/ FVC ratio. Median, (IQR)	**46.4 (36.8–67.4)**	**52.0 (41.5–61.6)**	**78.9 (72.7–81.9)**	< 0.0001*
GOLD classification, n (%)				
Normal spirometry GOLD Mild GOLD Moderate GOLD Severe GOLD Very severe	54 (23.7%) 13 (5.7%) 56 (24.7%) 74 (32.5%) 31 (13.6%)	0 6 (12%) 19 (38%) 21 (42.0%) 4 (8.0%)	51 (100%)	0.2922**
Kco, % predicted. Median (IQR)	**59.9 (48.3–74.9)**	**62 (47–77)**	**100.5 (82.5–109.75)**	< 0.0001*
Residual volume, % predicted. Median, (IQR)	**108.7** **(88.8–140.3)**	**117.5** **(100.5–145)**	**77.0** **(72.0–88.5)**	< 0.0001*
Reported exacerbation frequency in past 12 months				
Median (IQR) Frequent (2 or more), n (%) Infrequent (1), n (%) None, n (%)	**1 (0–2)** **82 (36.0%)** **52 (22.8%)** **94 (41.2%)**	**1.5 (1–3)** **26 (52%)** **14 (28%)** **10 (20%)**	N/A	0.0057*
Presence of Chronic Bronchitis	41(18.0%)	26 (52%)	0	< 0.0001**
Presence of emphysema on CT CT scans available (n) Emphysema present (n (%)	167 94 (56.3%)	50 23 (46.0%)	N/A	0.195**
COPD Assessment Test. Median (IQR)	**17 (12–23)**	**25 (21–28)**	n/a	< 0.0001*
Inhaled corticosteroid, n (%)	109 (48%)	25 (50%)	0	0.876**
Long-acting inhaled beta 2 agonist	148 (65%)	30 (59%)	0	0.249**
Long-acting inhaled anti-muscarinic	162 (71%)	37 (74%)	0	0.132**
BMI. Median (IQR) kg/m2	25 (23–28)	24 (23–26)	25 (23–29)	0.879*
Heart rate, bpm. Median (IQR)	**65 (58–72)**	**71 (62.3–79.0)**	**64 (57–72)**	0.003*
Systolic blood pressure, mmHg. Median (IQR)	**135 (126–151)**	**142 (131–155)**	**130 (124–138)**	0.0174*
Diastolic blood pressure, mmHg. Median (IQR)	81 (74–88)	79 (72–86)	78 (70–84)	0.08*
aPWV, m/s Median (IQR)	**9.4 (8.3–11.0)**	**10.0 (9.4–12.0)**	**8.3 (7.2–9.4)**	< 0.0001*
QRISK2**®** Score. Median (IQR) n = (excluding those with CVD)	**9.5 (4.1–15.1)** **n = 211**	**25 (17–32)** **n = 41**	**12.7 (9.8–25.4)** **n = 47**	< 0.0001*

Patient demographics as collected on the day of cardiovascular risk assessment. PiZZ, and PiZ “Null” are the deficiency alleles present in this patient population. Lung function measures are post bronchodilation and include standard measures of spirometry (FEV_1_/FVC) as well as measures of transfer coefficient (Kco). AATD and COPD patients were stratified in accordance with GOLD groups

BMI = Body Mass Index. QRISK2**®** Score was only calculated in those without a known history of CVD or a CVA as confirmed by self-report and General Practitioner records

*Kruskal Wallis

**Contingency (Fishers Exact or Chi squared). Bold results denote significant differences. Where significant differences were found across the three groups, Dunn’s multiple comparisons tests were performed between individual groups to determine which of these were significantly different. The significance values of these are described within the main body of the results

**Table 2:** Factors which make up other elements of the QRISK2® score

	AATD	Non-AATD COPD	Healthy controls	p value
Diabetes status				0.1771
None	217 (95%)	45 (90%)	44 (86%)	
Type 1	2 (0.9%)	1 (2%)	1 (2%)	
Type 2	9 (4%)	4 (8%)	6 (12%)	
Angina or heart attack in a first degree relative < 60 years	13 (6%)	4 (8%)	3 (6%)	0.627
Chronic kidney disease	2 (0.9%)	1 (2%)	0	
Atrial fibrillation	5 (2%)	1 (2%)	0	
Rheumatoid arthritis	1 (0.4%)	0	0	
Treated for hypertension	50 (22%)	13 (26%)	6 (12%)	0.174

Demographic data was collected for the QRISK2**®** score and compared across groups. Comparisons could only be made where sufficient numbers were included. Differences were compared with Fisher Exact tests and Chi squared

There were differences in the ages across the cohorts (Kruskal Wallis, p < 0.0001), with AATD patients being younger than both HC and COPD patients using a Tukey’s Multiple comparison test (p < 0.0001 for both). There were no differences in sex or in ex-smokers, but there were fewer never smokers in the COPD group, based on inclusion criteria. There were differences in pack year history between groups, with AATD patients having smoked less than both HC and non-AATD COPD.

Healthy controls had normal lung function across all physiology measures. There were no differences between absolute forced expiratory volume in one second (FEV_1_) or FEV_1_ percent predicted (Dunn’s Multiple Comparison, p = 0.06 and > 0.99 respectively), distribution of cases across Global Initiative for Chronic Obstructive Lung Disease (GOLD) disease severity stages or carbon monoxide transfer coefficient (K_CO_) and residual volume (Dunn’s multiple comparison test for both, p > 0.99 for both) between the AATD and non-AATD COPD groups.

### CVD risk tools provide concordant results in COPD and HC, but not AATD

Seventeen AATD patients (7.5%), 9 (18%) non-AATD COPD and 4 (7.8%) HC had a previous diagnosis of CVD, respectively. These were excluded from QRISK2® calculation, leaving 211 AATD, 41 COPD and 47 HC for comparisons of QRISK2® and aPWV.

**Fig. 1:**
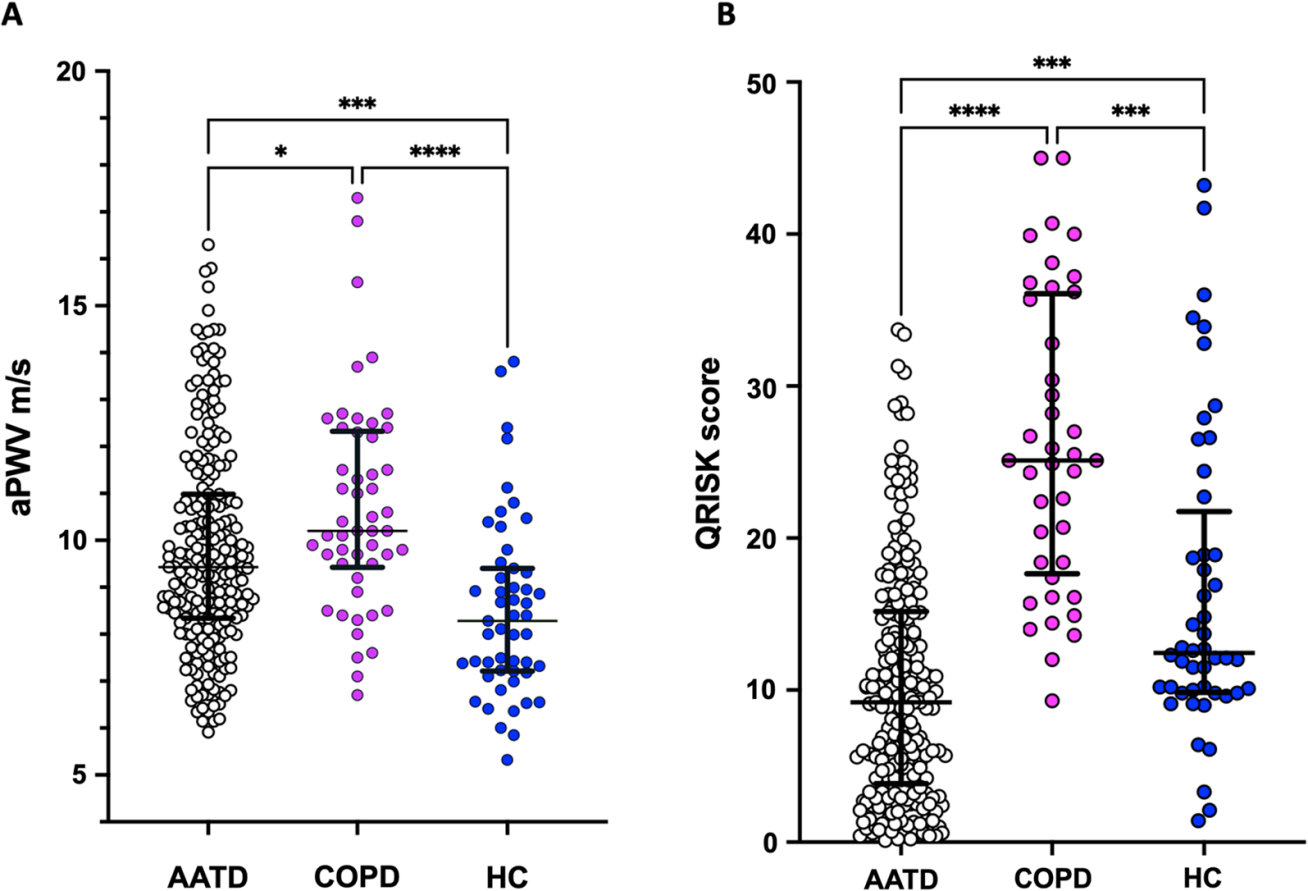
Pulse wave velocity and QRISK2® score. aPWV (**A**) and a QRISK2**®** score (**B**) was calculated per participant as described in the methods at the study visit using provided demographic and laboratory measures. In this figure the aPWV is unadjusted for age or smoking history, while QRISK2® automatically adjusts for these factors. QRISK2**®** score does not include those with known CVD. Each dot represents one measure. Median and IQR are shown. For both graphs, groups were compared with a Kruskal–Wallis test and Dunn’s Multiple Comparison test was then performed. **A**; * = p = 0.0225, *** = p = 0.0003, **** = p < 0.0001. For **B**; AATD vs HC p = 0.0005, COPD vs HC p = 0.0004, **** = p < 0.0001

When unadjusted aPWV readings were examined, there were differences between groups. Unadjusted aPWV was highest in non-AATD COPD and lowest in HC. See Fig. 1A. There were also differences in QRISK2®**®** score, which was highest in non-AATD COPD and lowest in AATD patients. See Fig. 1B.

In both COPD and HC groups, results were highly concordant between QRISK2® and aPWV, with no COPD patients or HC having a low QRISK2® score and a high aPWV or visa versa. The pattern was different for AATD patients. Here, while 116 people (55%) had concordant QRISK2® and aPWV results, 95 (45%) of people had discordant results, suggesting QRISK2® and aPWV were not aligning in this population in the same way as in the non-AATD COPD or HC cohorts.

The prevalence of the co-morbidities and family history listed in the QRISK2® were assessed to understand if these could account for differences in risk between the three cohorts of people. There were no other differences in co-morbidities or family history used to calculate QRISK2® score. See Table 2. This suggests the higher QRISK2**®** score in non-AATD COPD and HC compared to AATD patients reflected their increased age and smoking history. QRISK2® automatically adjusts for age and smoking status. Although aPWV increases with both age and pack year history, there is no adjustment for these factors in the raw measurements. As the AATD group were both younger and had smoked less than the COPD and HC cohorts, multivariate analysis was undertaken to determine if aPWV was higher in AATD once age and smoking history were taken into account.

### CVD risk using aPWV is higher in AATD compared to non-AATD COPD and HC when age and smoking are corrected for

In a multivariable regression model which adjusted for age and smoking history (total model p < 0.0001, Adjusted R^2^ = 0.23), aPWV was lower in both non-AATD COPD (co-efficient − 0.90 (95% CI − 1.65 to − 0.14, p = 0.02) and HC (co-efficient − 1.84 (95% CI − 2.60 to − 1.11, p < 0.0001) compared with AATD.

### There are relationships between CVD risk as measured by aPWV and lung function in AATD, but not QRISK2®

When groups were combined, there were weak inverse relationships between aPWV and K_CO_ percent predicted (rho = − 0.371, p < 0.0001), FEV_1_ percent predicted (rho = − 0.273, p < 0.0001) and FEV_1_/ forced vital capacity (FVC) ratio (rho =—0.265, p < 0.0001). However, there were no such relationships between QRISK2**®** score and lung function (FEV_1_ percent predicted (rho = − 0.054, p = 0.345), FEV_1_/FVC ratio (rho = 0.053, p = 0.353) and K_CO_ percent predicted (r = 0.052, p = 0.371).

The relationships between lung physiology and aPWV and QRISK2**®** were assessed in the AATD group alone. FEV_1_ and K_CO_ strongly correlated with each other and were not modelled together but were entered into the models separately. In multivariable regression there were relationships between K_CO_ percent predicted, age, ever smoking status and aPWV (R [2] − 0.35, p < 0.001). There were also relationships between FEV_1_ percent predicted, age, ever smoking status and aPWV (R [2] − 0.25, p < 0.001).

There were no relationships between lung physiology and QRISK2**®** in AATD or non-AATD COPD.

### There are relationships between arterial stiffness and emphysema in AATD but not QRISK2®

Ninety-four AATD patients had visible emphysema on CT and 73 did not.

Those with emphysema had a higher aPWV (10.3 (9.3–13.1) m/s Vs. 8.9 (8.2–10.4) m/s, p < 0.0001). There was no differences in QRISK2**®** score between those with emphysema and those without (10.2 (4.9–16.4) Vs. 10.2 (3.3–14.7), p = 0.352).

There were no differences in age between these subgroups (59.4 years (50.12–65.8) Vs. 58.9 (49.9–66.9), p = 0.880) but those with emphysema had smoked more, (18.9 pack-years (10.4–25.0) Vs. 7.5 (1.1–15.0), p < 0.0001). Data is median and IQR throughout and compared using Mann Whitney tests.

### There is a relationship between neutrophil proteinase activity and CVD risk, measured using aPWV, in AATD

**Fig. 2:**
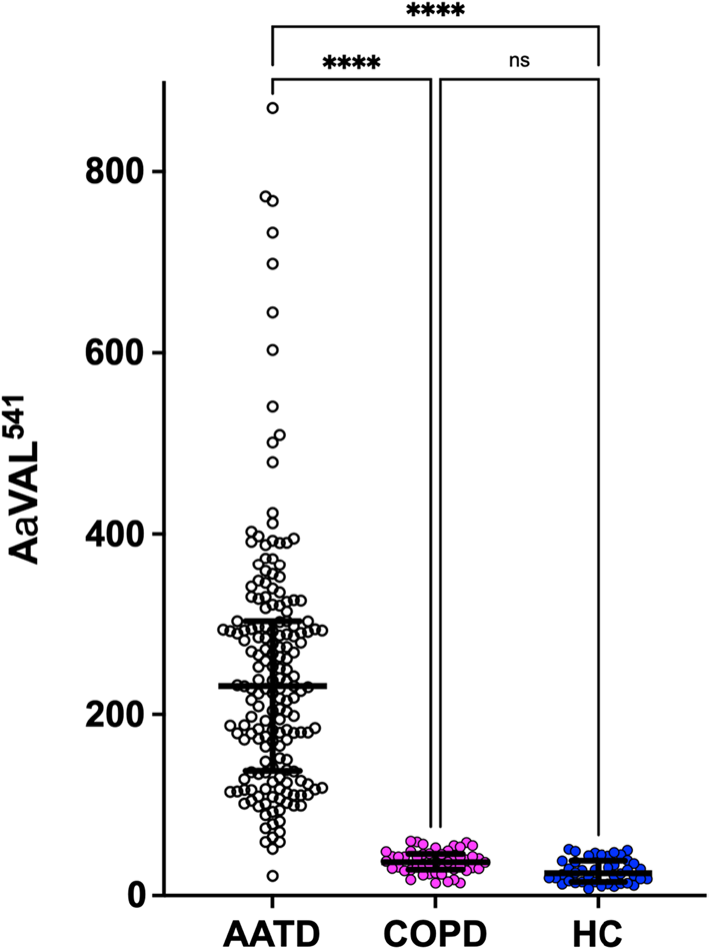
Proteinase 3 activity footprint in AATD, non-AATD COPD and HC. AαVAL^541^ is measured in nM. Each dot represents one person’s measurement, taken from a plasma sample. Results were compared initially using Kruskal Wallis and then Dunn’s Multiple comparison. **** = p < 0.0001

AATD, non-AATD COPD and HC patients from Birmingham, had bloods taken to assess the proteinase 3 activity footprint. As described [26], AαVAL^541^ was higher in AATD than non-AATD COPD and HC. See Fig. 2.

**Fig. 3:**
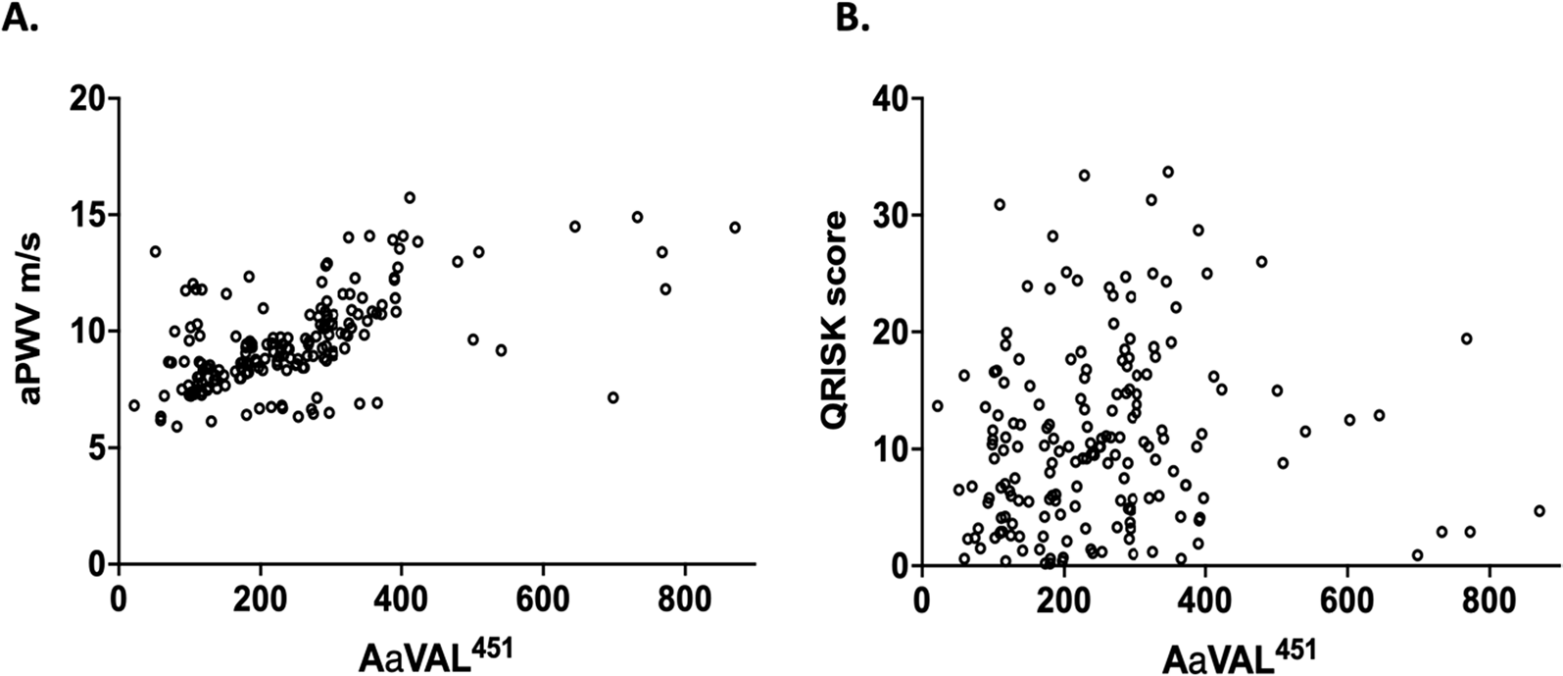
The relationship between AαVAL^541^ and aPWV or QRISK2® score in AATD. AαVAL^541^ is measured in nM**.** Each dot represents one person’s data. Spearman’s Correlation was applied. Although both aPWV and QRISK2**®** related to the proteinase 3 activity footprint, the relationship with aPWV was stronger (Rho 0.5724 (p < 0.0001) vs. Rho 0.1876 (p = 0.0124), respectively)

For AATD patients, there was a moderately strong relationship between AαVAL^541^ and aPWV (Rho = 0.572, p < 0.0001) and a weak relationship between AαVAL^541^ and QRISK2® (Rho = 0.188, p = 0.0124). See Fig. 3A, B respectively.

There were no such relationships with non-AATD COPD: AαVAL^541^ and aPWV (Rho = 0.245, p = 0.089) and QRISK2® (Rho = 0.213, p = 0.145) respectively; or HC: AαVAL^541^ and aPWV (Rho = − 0.267, p = 0.092) and QRISK2® (Rho = − 0.182, p = 0.266) respectively.

A multivariate regression analysis was performed, studying CVD risk and proteinase 3 activity, adjusting for age and smoking status. In AATD patients, with adjustments for age and never or ever smoking (total model p < 0.0001, Adjusted R [2] = 0.33) there remained a relationship between AαVAL^541^ and aPWV (co-efficient 43.6, 95% CI 34.2 to 53.1, p < 0.0001). This was also true when pack years of smoking and age were included in the model (total model p < 0.0001, Adjusted R^2^ = 0.33), co-efficient 43.2, 95% CI 33.8 to 52.6, p < 0.0001).

Also, there remained a relationship between AαVAL^541^ and aPWV even after adjustment for QRISK2®, age, and smoking status (ever or never; total model p < 0.0001, Adjusted R [2] = 0.32, co-efficient 43.6, 95% CI 34.7 to 53.13, p < 0.0001: pack year history; total model p < 0.0001, Adjusted R [2] = 0.33, co-efficient 43.2, 95% CI 33.7 to 52.6, p < 0.0001).

There were no relationships between AαVAL^541^ and aPWV in non-AATD COPD when smoking status and age were included (ever smoking and age; total model p = 0.1778, adjusted R [2] = 0.04, co-efficient 1.4, 95% CI − 0.34 to 3.16: Pack year and age; total model p = 0.3081, adjusted R [2] = 0.019, co-efficient 1.29, 95% CI − 0.47 to 3.05). There was no relationship between AαVAL^541^ and aPWV in non-AATD COPD when QRISK2® was included (ever or never; total model p < 0.292, Adjusted R [2] = 0.023, co-efficient 1.33, 95% CI − 0.53 to 3.19: pack year history; total model p < 0.459, Adjusted R [2] = 0.007, co-efficient 1.20, 95% CI − 0.71 to 3.11).

### Longitudinal follow up and outcomes

AATD, COPD and HC participants in Birmingham were followed up after 4 years (180 patients with AATD, 50 with COPD and 50 HC) for any new diagnosis of CVD. In total there were 23 AATD, 18 COPD and 7 HC participants with a new diagnosis of CVD, respectively. See Table 3. Across all groups, a new diagnosis of CVD during this follow up was associated with a higher baseline QRISK2**®** and aPWV but patients with a new diagnosis of CVD and AATD were younger, had smoked less and had a lower QRISK2® score than patients with COPD or HC with CVD. Statistical analysis comparing incidence of new diagnosis of CVD was not performed as this would not account for differences in age and smoking status between groups, which are important independent risk factors for CVD.

**Table 3:** Medical diagnoses of CVD after 4 years follow-up

	AATD		Non-AATD COPD		Healthy Controls	
CVD	Yes	No	Yes	No	Yes	No
N	23	146	18	23	7	39
Age	61 (57–68)*^$^	56 (48–64)	74.0 (68–76)	70 (67–77)	69 (49–76)	69 (61–76)
PYH	10.5 (1.1–28.0)^$^	11.4 (15–20.8)	41.5 (36.3–55.0)	43.0 (26.0–51.0)	27.5 (19.7–32.5)	20.5 (10.5–42.3)
aPWV	11.1 (9.9–12.8)*	8.8 (8.0–9.8)	11.4 (10.1–12.6)*	9.7 (8.4–11.0)	10.5 (8.9–11.7)	8.6 (7.22–11.8)
QRISK2**®**	19.4 (8.1–25.0)*^$^	9.5 (4.2–13.8)	31.8 (26.0–37.2)*	18.5 (14.9–25.1)	33.9 (28.2–35.3)*	16.9 (6.1–24.2)

Medical notes of all participants were reviewed 4 years after baseline visit. Diagnoses of CVD was physician confirmed. CVD included angina, a myocardial infarction or stroke. Numbers exclude those with a diagnosis of CVD at baseline. All data is presented as median with IQR apart from numbers, which are absolute numbers

*Statistically different from same patient group without CVD

^$^AATD statistically different from non-AATD COPD and HC when all patients with CVD were compared

PYH = pack year history

## Discussion

The current paper highlights four important points.

First, CVD risk, as measured using the physiological marker of arterial stiffness, and adjusted for smoking status, sex and age, is increased in AATD compared with non-AATD COPD or healthy controls.

Second, unlike patients with non-AATD COPD and HC, there was significant difference in CVD risk assessment using aPWV and QRISK2**®** in AATD**.** This discordance was not seen in HC or patients with non-AATD COPD. This questions whether QRISK2® is the right risk assessment tool if used in isolation in AATD. The longitudinal assessment of CVD in the current study also supports this, with AATD patients who went on to develop CVD being younger, having smoked less and having a lower QRISK2**®** than healthy controls or patients with non-AATD COPD.

Third, there were relationships between aPWV and K_CO_ and FEV_1_ in AATD, which persisted after adjusting for age and smoking status. This supports the concept of shared processes contributing to both lung disease and arterial stiffness in AATD.

Fourth, as expected, proteinase 3 activity, intrinsically linked with lung damage in AATD [28], was highest in AATD. Furthermore, proteinase 3 activity was also associated with aPWV in AATD, even following adjustment for traditional CVD risk factors. There were relationships between proteinase 3 activity and aPWV, which remained in a multi-variate regression model which included age, smoking status, and QRISK2® score. This provides evidence to support a potentially causative association between CVD and neutrophil proteinases in AATD.

The QRISK2® score adjusts for traditional risk factors for CVD and was on average lowest in patients with AATD compared with non-AATD COPD and HC, while the age and smoking adjusted aPWV was highest in the AATD cohort. Previous studies assessing CVD risk in AATD have shown contradictory results, but most studies have been small or have included patients on augmentation therapy [29]. The European AATD Research Collaboration international registry suggested that in untreated homozygous Z patients’ the prevalence of cardiovascular was 20.9%, which was even higher in augmented patients (38.5%) [19]. Although the reasons are currently unknown, this could reflect differences in disease severity, progression rates or co-morbidities [19] which were main factors in deciding on augmentation therapy. A Danish study of only 6 ZZ homozygotes and 39 MZ heterozygotes described lower blood pressure with MZ patients having a lower risk of coronary vascular disease compared to non-AATD controls [15]. A study of 139 AATD patients found those already receiving augmentation therapy (n = 110) had a lower blood pressure and a lower prevalence of diagnosed CVD, (taking into account age, smoking, body mass index and lung function), compared to non-AATD COPD [29]. Other studies have suggested a higher potential risk of CVD by aPWV including one of 19 AATD patients and 20 age-matched controls [21]. Arterial stiffness was also raised in 33 patients with AATD [17] and a study of 51 AATD patients showed evidence of large vessel degeneration [22].

The study presented here is the largest and most comprehensive study of patients with confirmed and non-augmented AATD (PiZ and Znull). The discordance between cardiovascular risk in AATD using QRISK2® and aPWV is an important finding. In usual clinical practice, QRISK2**®** is frequently used for sole CVD risk assessment, but unlike in non-AATD COPD and the HC in this study, QRISK2**®** did not identify a significant proportion of patients with a raised aPWV in AATD (perhaps because of a lower smoking history). This suggests that traditional CVD risk factors may not account for all CVD in AATD and that QRISK2® alone may not be a sufficient screening tool to detect CVD in AATD.

The discordance between cardiovascular risk in AATD as measured by aPWV (especially when adjusted for age and smoking status) and QRISK2® would support the premise that traditional risk factors may not account for, and therefore, might not identify, all CVD risk in AATD.

In non-AATD COPD there is a strong relationship between QRISK2® score and aPWV, retained in multivariate analysis [30]. This is also true for asymptomatic healthy controls, where QRISK2**®** scores and aPWV align [31]. If CVD risk scores give discordant results in AATD whilst, they correlate in other diseases, it is important to understand the actual risk of CVD and the mechanism for risk including the best non-invasive tool to predict risk and the best measures to reduce this risk including augmentation therapy in studies described above.

The use of QRISK2® in AATD may systematically underestimate measured CVD risk, although if aPWV is high and QRISK2® is low it raises issues about relevant preventative management. The current paper highlights the relationship between neutrophil proteinases and arterial stiffness in AATD, suggesting proteinases as a potential therapeutic target and hence augmentation as a possible strategy to reduce risk.

There is a wealth of evidence supporting the role of the neutrophil in AATD, with cell numbers and their products mechanistically linked to all aspects of disease [27]. Augmentation therapy has been the cornerstone of specific treatment of AATD for decades, with studies showing that it reduces the rate of progression of emphysema [4]. Coronary and large arterial blood vessels contain elastin. When elastin fibres are damaged, contraction is transferred to collagen fibres, which are 100–1000 times stiffer than elastin fibres [32]. Proteinase activity is associated with arterial stiffness measured by aPWV [33] and there is an increasing body of evidence implicating neutrophils, and specifically proteinases in the pathogenesis of CVD (recently reviewed [34]).

If neutrophil proteinases are implicated in CVD, it is logical to assume the signal will be amplified in AATD patients who are not receiving augmentation therapy, and that the signal could be reduced in AATD patients receiving augmentation therapy This would be consistent with the current study and studies of CVD in AATD reported to date (and described above). It is possible that augmentation therapy may therefore reduce proteinase-associated CVD risk in AATD. Indeed the data presented here suggests, a study of proteinase-directed therapy to reduce CVD risk in AATD should be undertaken.

The current study has strengths. It includes the highest number of unaugmented AATD patients involved in a study of CVD risk, and so represents the natural history of the disease. It includes validated tests to assess CVD risk, a validated marker of proteinase 3 activity and lung function performed by trained physiologists to international standards. All tests were performed, and samples collected on the same day. Longitudinal follow up enabled assessment of subsequent emergence of new diagnoses of CVD.

Statistical adjustment was performed to minimise the confounding effects of traditional CVD risk factors, such as age and smoking history. However, the relationship between proteinase activity and aPWV was maintained in AATD patients even after this adjustment. Although adjustment of age and smoking history changed the relationship of aPWV values between groups, with aPWV becoming higher in the AATD group than non-AATD COPD patients and HC. This suggests that AATD alone accounts for the increased risk in this patient cohort.

The current study has some potential weaknesses. It did not include coronary artery angiograms to diagnose CVD definitively in AATD but instead measured or calculated CVD risk. However, patients were followed up to assess the development of CVD over time. Formal longitudinal studies will be needed to determine whether the degree of neutrophil activity (which can be variable in AATD) influences the severity of CVD or the timing of its acquisition, but this type of study will always be challenging in a rare disease. The numbers of usual COPD and HC needed for the current study were based on aPWV for power calculations, and other comparisons may remain underpowered and interpretation should be considered with this in mind. Patients were recruited from secondary care clinics, research cohorts and specialist clinics, and therefore may reflect a selection bias. No adjustments were undertaken for multiple comparisons, but exact p values are provided.

## Conclusions

In conclusion, this study provides important insights into CVD risk in AATD, highlighting elevated risk measured by arterial stiffness when adjusted for age and smoking history and is not captured by QRISK2®. The study supports using physiological tests (such as aPWV) when screening for CVD risk in AATD, and not relying on traditional risk factors alone.

This study suggests relationships between CVD and lung disease in AATD, with a proposed mechanism being proteinase-driven breakdown of elastin fibres in the large arteries and lungs. Studies assessing the effects of augmentation therapy or anti-proteinases on CVD in carefully matched AATD patients are now indicated, to test this hypothesis further.

## Methods

This observational study was approved by the Health Research Authority and National Research Ethics Committees (REC) and all research procedures took place after gaining informed written consent.

Three cohorts of participants were recruited in Birmingham, UK; the AATD registry (REC:3359a), the West Midlands COPD database (REC:12/EM/0090) and a cohort without COPD or AATD (termed healthy controls (HC)) (REC:15/WM/0002). Some AATD patients were recruited from the Royal Free London NHS Foundation Trust (RFH) (REC:13/LO/1085).

All participants were recruited between 2016 and 2018. Participants medical notes were screened 4 years after recruitment to assess for evidence of emergent CVD.

Inclusion criteria were as follows:

*AATD*:

1. Confirmed AATD with a serum α_1_-antitrypsin (ATT) < 7 µM
2. A severe deficient phenotype/genotype (PiZ, Znull} confirmed by isoelectric focusing and specific gene primers for Z S and M sequences (Heredilab, Salt Lake City, UT, USA).
3. Provision of informed consent
4. Never received augmentation therapy for AATD
5. No immunosuppressive drugs including oral corticosteroids
6. No exacerbation in the preceding 8 weeks
7. No lung surgery or transplantation

*COPD*:

1. No evidence of AATD with a serum α_1_-antitrypsin (ATT) > 20 µM
2. Confirmed diagnosis of COPD (based on GOLD criteria with fixed FEV_1_/FVC ratio).
3. Active or past smoking history with > 10 pack year history
4. Provision of informed consent
5. No immunosuppressive drugs including oral steroids
6. No exacerbation in the preceding 8 weeks
7. No lung surgery or transplantation

*Heathy controls*:

1. No evidence of AATD with a serum α_1_-antitrypsin (ATT) > 20 µM
2. Normal spirometry and no chronic lung disease confirmed by a respiratory consultant
3. Provision of informed consent
4. No immunosuppressive drugs including oral steroids
5. No chest infections in the preceding 8 weeks

Patients were selected sequentially at follow up attendance to reduce selection bias. A detailed medical history (with comorbidities confirmed by general practitioner where possible) was documented.

Measures of lung function were undertaken by trained respiratory physiologists in accordance with national guidelines [35]. All tests were conducted in the stable state, post bronchodilator. Lung-volume measurements were assessed using helium dilution (Morgan Medical, Kent, UK) and gas transfer by the single-breath carbon monoxide method. Lung function values were expressed using percent predicted values based on the Global Lung Function Initiative (GLI) reference ranges [36].

All non-AATD COPD and 73% of the AATD patients had Computed Tomography (CT) scans within the last 4 years which were reviewed for visible emphysema (reported as present or not, confirmed by both a respiratory consultant and a consultant radiologist).

The AAT phenotype and genotype were assessed on dried blood spots by Heredilab Inc, 1060 E 100 S Ste 109 Salt Lake City, UT, 84102-4303 United States. All were PiZ on isoelectric focussing (IEF) (only Z specific bands seen) and the majority only had copies of the Z allele using Z specific primers. 7 were positive for Z, but although the M specific sequence was also detected, the low blood concentration (< 7µM) and absence of M band seen on IEF suggested a non-translated gene and in the absence of specific primers and whole gene sequencing these were labelled PiZ “null”.

### CVD risk

Aortic stiffness was assessed using the carotid-femoral pulse wave velocity as previously described [37] prior to bronchodilation. Studies were conducted using the Vicorder**®** device, (SMT Medical, Germany). Here, an aPWV > 10m/s was considered high [38]. Blood pressure (BP) and heart rate were measured with an oscillometric device on the dominant arm. Mean arterial pressure (MAP) was calculated from systolic BP and diastolic BP as MAP = [(2 × diastolic) + systolic]/3.

QRISK2® score was calculated in patients without a history of cardiovascular disease using a freely available tool (www.qrisk.org), as described with < 10% being considered a low risk and > 20% being considered a high risk of CVD [39].

### Neutrophil proteinase activity

Aα-Val^541^, a proteinase 3 specific fibrinogen degradation product and a surrogate marker of neutrophil degranulation in vivo, was measured in plasma as described previously [26] from a sample drawn at the same visit as lung function and aPWV measurements.

### Statistical analysis

A difference in aPWV 2m/s (standard deviation 2.4m/s) has been reported in other group comparisons and was used to perform power calculations [21]. A larger group of AATD patients was recruited, based on pragmatic patient availability. Statistical analyses were performed using STATA Statistics, (StataCorp. 2019. Stata Statistical Software: Release 16. StataCorp LLC.). Variables are presented as stated. Data distribution was assessed both visually and using the Kolmogorov–Smirnov test. Analysis methodology is stated in text. In multivariable regression a backwards stepwise approach was used with consideration for biological plausibility and a conservative significance level of p < 0.1 was utilised as inclusion criteria in the final model. No adjustments to significance (p) values were made for multiple comparisons, but exact p values are provided.

## Data Availability

All data is stored on a University encrypted database. The datasets are not publicly available and can solely be accessed by the authors as outlined on the signed patient consent forms. However, datasets used and/or analysed during the current study are available from the corresponding author on reasonable request.
